# Long‐term persistence of wildlife populations in a pastoral area

**DOI:** 10.1002/ece3.6658

**Published:** 2020-08-07

**Authors:** Christian Kiffner, John Kioko, Jack Baylis, Camille Beckwith, Craig Brunner, Christine Burns, Vasco Chavez‐Molina, Sara Cotton, Laura Glazik, Ellen Loftis, Megan Moran, Caitlin O'Neill, Ole Theisinger, Bernard Kissui

**Affiliations:** ^1^ Center for Wildlife Management Studies The School For Field Studies Karatu Tanzania; ^2^ Department of Environmental Studies and Sciences Santa Clara University Santa Clara CA USA; ^3^ Biology Department Wheaton College Norton MA USA; ^4^ Psychology Department Whitman College Walla Walla WA USA; ^5^ Department of Environmental Science Dickinson College Carlisle PA USA; ^6^ Department of Environmental Studies College of the Holy Cross Worcester MA USA; ^7^ Neuroscience and Behavior Department Vassar College Poughkeepsie NY USA; ^8^ Department of Animal Science University of Illinois, Urbana‐Champaign Champaign IL USA; ^9^ Rubenstein School of Environment and Natural Resources University of Vermont Burlington VT USA; ^10^ Biology Department College of the Holy Cross Worcester MA USA; ^11^ Department of Biology St. Mary's College of Maryland St. Mary's City MD USA

**Keywords:** coexistence, competition, conservation effectiveness, evidence‐based conservation, facilitation, population dynamics

## Abstract

Facilitating coexistence between people and wildlife is a major conservation challenge in East Africa. Some conservation models aim to balance the needs of people and wildlife, but the effectiveness of these models is rarely assessed. Using a case‐study approach, we assessed the ecological performance of a pastoral area in northern Tanzania (Manyara Ranch) and established a long‐term wildlife population monitoring program (carried out intermittently from 2003 to 2008 and regularly from 2011 to 2019) embedded in a distance sampling framework. By comparing density estimates of the road transect‐based long‐term monitoring to estimates derived from systematically distributed transects, we found that the bias associated with nonrandom placement of transects was nonsignificant. Overall, cattle and sheep and goat reached the greatest densities and several wildlife species occurred at densities similar (zebra, wildebeest, waterbuck, Kirk's dik‐dik) or possibly even greater (giraffe, eland, lesser kudu, Grant's gazelle, Thomson's gazelle) than in adjacent national parks in the same ecosystem. Generalized linear mixed models suggested that most wildlife species (8 out of 14) reached greatest densities during the dry season, that wildlife population densities either remained constant or increased over the 17‐year period, and that herbivorous livestock species remained constant, while domestic dog population decreased over time. Cross‐species correlations did not provide evidence for interference competition between grazing or mixed livestock species and wildlife species but indicate possible negative relationships between domestic dog and warthog populations. Overall, wildlife and livestock populations in Manyara Ranch appear to coexist over the 17‐year span. Most likely, this is facilitated by existing connectivity to adjacent protected areas, effective anti‐poaching efforts, spatio‐temporal grazing restrictions, favorable environmental conditions of the ranch, and spatial heterogeneity of surface water and habitats. This long‐term case study illustrates the potential of rangelands to simultaneously support wildlife conservation and human livelihood goals if livestock grazing is restricted in space, time, and numbers.

## Graphical abstract

To provide evidence for livestock–wildlife coexistence, we present data on long‐term (intermittently from 2003 to 2008 and regularly from 2011 to 2019) monitoring and population dynamics of livestock and wildlife species in a managed pastoral area in northern Tanzania. Our results show constant livestock population trajectories and constant to increasing wildlife population trends. This case study illustrates the potential of rangelands to simultaneously support wildlife conservation and human livelihood goals if livestock grazing is managed sustainably.

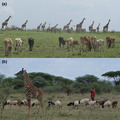

## 1 INTRODUCTION

Wildlife populations on the African continent are under substantial anthropogenic pressure and experience marked population declines particularly in Central, Western, and Eastern Africa (Craigie et al., 2010). In East Africa, wildlife populations have declined in national parks as well as in multiple‐use areas which allow some form of natural resource utilization (Caro, 1999; Mtui, Owen‐Smith, & Lepczyk, 2017; Ogutu, Kuloba, Piepho, & Kanga, 2017; Ogutu, Owen‐Smith, Piepho, Kuloba, & Edebe, 2012; Stoner et al., 2007; Western, Russell, & Cuthil, 2009). Several factors have been hypothesized to cause these widespread wildlife population declines. These can be summarized in unsustainable (legal or illegal) hunting of wildlife, and habitat loss and fragmentation due to land use changes (Caro & Scholte, 2007; Fynn & Bonyongo, 2011; Kiffner et al., 2017; Lindsey et al., 2013; Newmark, 2008; Ogutu et al., 2012; Ripple et al., 2015, 2016). Concurrent livestock population increases have also been associated with declines of wildlife populations (Gordon, 2018; Homewood, Trench, & Brockington, 2012; Ogutu et al., 2016; Prins, 1992). The livestock–wildlife interface plays a complex and controversial role in this debate. On the one hand, unfenced pastoral areas can ensure wildlife movements between protected areas and thus effectively prevent isolation of protected areas (Western et al., 2020), but on the other hand livestock can negatively affect wildlife populations (Schieltz & Rubenstein, 2016).

Livestock grazing can cause wildlife declines via multiple mechanisms. Livestock may reduce overall food availability to wildlife by foraging on (and thus reducing) shared and limited resources and this form of competition may be of particular importance during the dry season when resources are scarce (Odadi, Jain, Van Wieren, Prins, & Rubenstein, 2011; Odadi, Karachi, Abdulrazak, & Young, 2011). Additionally, severe grazing pressure can cause soil erosion which subsequently can reduce vegetative cover and primary productivity of the area (Butt & Turner, 2012; Hitchcock, 2000; Kimuyu et al., 2017). Livestock grazing may also alter vegetation structure and fire regimes, which can ultimately homogenize habitat structure (Hempson, Archibald, & Bond, 2017; Veldhuis, Ritchie, et al., 2019). In addition, it has long been established that livestock species can introduce and transmit parasites and pathogens to wildlife (Craft, 2015; Roeder, Mariner, & Kock, 2013). Additionally, domestic dogs associated with livestock may directly (via predation) and indirectly (via risk effects) negatively influence wildlife populations (Doherty et al., 2017; Gompper & Vanak, 2008; Vanak & Gompper, 2009).

However, livestock–wildlife interactions are not exclusively associated with negative outcomes for wildlife populations, and under certain circumstances can create conditions that are beneficial to wildlife populations. Indeed, multiple experimental and correlational studies indicate positive outcomes associated with livestock–wildlife coexistence. For example, livestock grazing can maintain and create habitat heterogeneity (Derner, Lauenroth, Stapp, & Augustine, 2009; Veblen & Young, 2010), facilitate grass growth during the growing season (Arsenault & Owen‐Smith, 2002; Odadi, Jain, et al., 2011; Odadi, Karachi, et al., 2011) and veterinary treatment of livestock can reduce overall parasite abundance and pathogen prevalence in the landscape (Grzeda et al., 2017; Keesing, Allan, Young, & Ostfeld, 2013; Keesing et al., 2018; Welsh, Keesing, & Allan, 2018).

These considerations suggest that livestock–wildlife interactions involve substantial trade‐offs and that wildlife and livestock can coexist if positive and negative direct and indirect livestock–wildlife interactions are managed strategically (Fynn, Augustine, Peel, & de Garine‐Wichatitsky, 2016; Keesing et al., 2018; du Toit, Cross, & Valeix, 2017; du Toit, Kock, & Deutsch, 2010). Although there are published examples indicating that East African rangelands can support diverse and abundant wildlife populations (e.g., Georgiadis, Olwero, Ojwang', & Romañach, 2007; Kinnaird & O’Brien, 2012; Ogutu et al., 2017; Rannestad, Danielsen, Moe, & Stokke, 2006; Schuette, Creel, & Christianson, 2016), few publications describe long‐term monitoring efforts and population dynamics of wildlife and livestock species in shared landscapes. Such long‐term monitoring is, however, required to objectively assess the long‐term net conservation outcome of the trade‐off between livestock and wildlife interactions in coupled social–ecological systems (Caughley, 1994; Danielsen, Burgess, & Balmford, 2005; Kremen, Merenlender, & Murphy, 1994; Newmark & Hough, 2000; Yoccoz, Nichols, & Boulinier, 2001). One reason for the scarcity of long‐term livestock and wildlife data in rangelands may be the fact that monitoring animal populations is relatively expensive and logistically challenging (Caro, 2016; Greene, Bell, Kioko, & Kiffner, 2017; Msoffe et al., 2010). For example, line distance sampling is widely used to estimate the density of ungulates and, ideally, requires a systematic transect layout to ensure that sampling locations are representative of the entire area allowing unbiased density estimation (Marques, Buckland, Bispo, & Howland, 2013; Thomas et al., 2010). Because implementing systematic transect designs with vehicles (due to off‐road restrictions, inaccessible terrain) or on foot (due to logistic or safety concerns) are challenging or impossible, many researchers monitor wildlife populations along road transects (e.g., Caro, 1999; Kiffner et al., 2020; Ogutu, Bhola, Piepho, & Reid, 2006). Quantifying potential bias associated with such nonrandom sampling would, however, allow for correcting of potential under‐ or over‐counting bias and thus provide greater credibility to density estimates.

**FIGURE 1:**
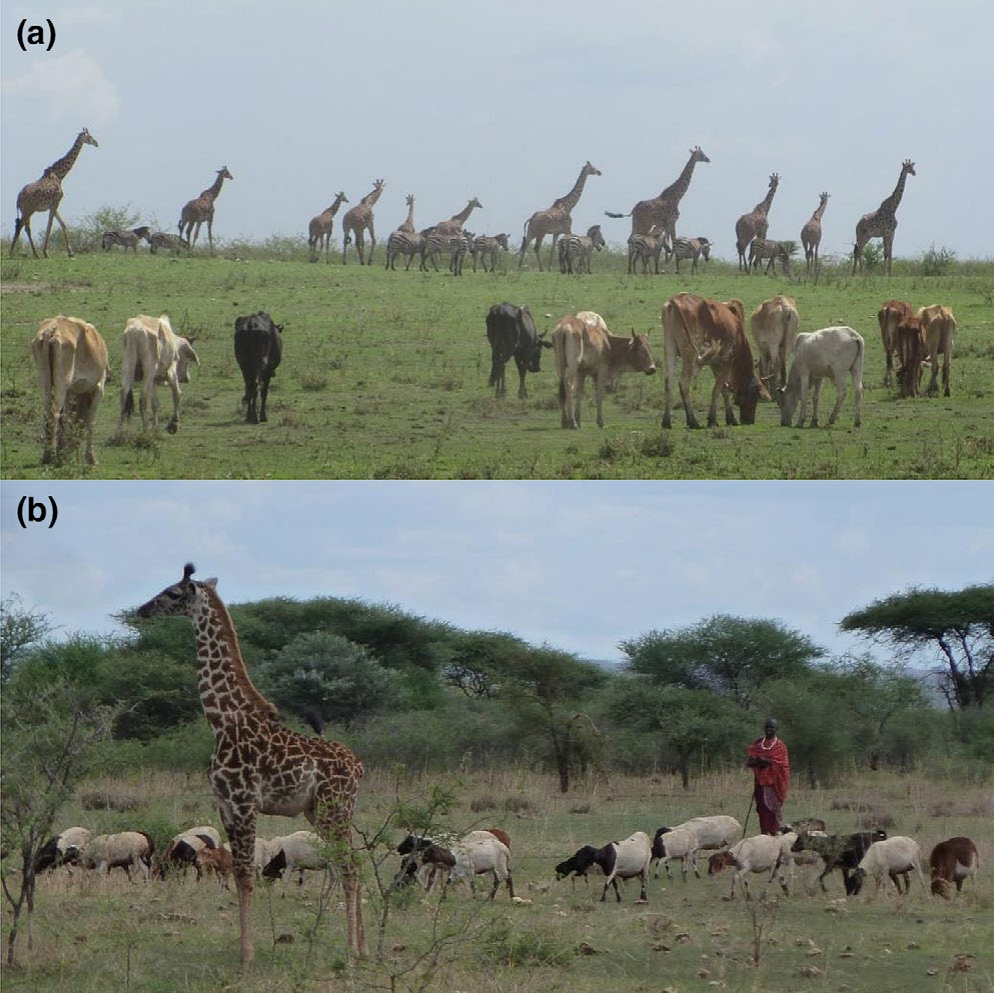
(a) Cattle grazing next to zebra and giraffe (Photo: John Kioko), and (b) sheep, herded by a Maasai, grazing next to juvenile giraffe in Manyara Ranch, northern Tanzania (Photo: Christian Kiffner)

Here we focus on one case study, Manyara Ranch in northern Tanzania. The management of the area follows a relatively unique conservation approach which aims at sustaining the pastoral lifestyle of adjacent *Maasai* communities as well as protecting wildlife populations (Figure 1; Sumba, Bergin, & Jones, 2005). We first present seasonal population density estimates of 14 wildlife and four livestock species (groups) based on road transect surveys conducted from 2003 to 2019 (occasionally from 2003 to 2008 and regularly from 2011 to 2019), test for potential design‐based biases of this monitoring approach and assess temporal trends of the surveyed animal populations. Furthermore, we test the hypotheses that interference competition between livestock and wildlife species is most evident (and manifested via negative population‐level effects) between livestock and wildlife species of similar feeding niches (Fritz, Garine‐Wichatitsky, & Letessier, 1996; Fynn et al., 2016; Voeten & Prins, 1999; Voeten, van de Vijver, Olff, & van Langevelde, 2010). Finally, we test the hypothesis that domestic dog population densities have negative population‐level consequences for wildlife species with a similar ecological niche (Black‐backed jackal *Canis mesomelas*) and for potential prey species.

## 2 MATERIALS AND METHODS

### 2.1 Study area

Manyara Ranch (MR) is an unfenced area, located in the Monduli district in northern Tanzania, approximately between −3.474° and −3.666° latitude and 35.936° and 36.079° longitude (Sumba et al., 2005). Rainfall is highly seasonal, mainly occurring during the long (March–May) and the short (November–December) rainy seasons and annual precipitation in the area ranges between 434 and 824 mm (Prins & Loth, 1988). MR is 182.98 km^2^ in extent and the vegetation is dominated by *Acacia‐Commiphora* bushland. The Makuyuni River flows through the ranch and several man‐made dams provide additional surface water access to wildlife and livestock species (Kioko, Zink, Sawdy, & Kiffner, 2013).

Historically, the area was a dry season grazing area of two adjacent *Maasai* communities (Esilalei and Oltukai). From 1956 to 1971, the area was—in economic agreement with the neighboring Maasai communities—managed by a German cattle farmer. From 1971 to 2001, the area was managed by the National Ranching Company (NARCO). The ranch did not operate profitably and the government sought to privatize it. From 2001 onwards, the Tanzanian Land Conservation Trust (a consortium managed by a board of trustees with representatives from conservation organizations, Tanzania National Parks, the local Maasai community, and the private sector) held the land title. In 2017, the oversight and management of MR were handed over to Monduli district. However, this change did not effectively change management objectives, practices, or personnel of MR. Since 2001, the management of MR has been supported by African Wildlife Foundation (AWF) (Rodgers, 2006). Since 2001, the key objective of MR “is to promote nature preservation and conservation and economic activities compatible with conservation for the benefit of present and future generations throughout Tanzania” (Sumba et al., 2005). No hunting is allowed, and the main income‐generating land uses are pastoralism and photographic tourism; operational costs are subsidized by AWF. Currently, MR keeps c. 800 Boran cattle (*Bos indicus*) and 400 Somali sheep (*Ovis aries*) (Warwick et al., 2016) and adjacent *Maasai* pastoralists are given spatially and temporally restricted grazing rights. Livestock from the community are allowed to graze only during the dry season using the 4 km buffer as their grazing area (and thus are not permitted to graze in the central area of the ranch). Rangers patrol the area and enforce anti‐poaching laws and grazing restrictions. Since 2013, Honeyguide Foundation, a nongovernmental organization specialized in anti‐poaching operations, manages and implements law‐enforcement activities in this area.

The area holds a central role in maintaining large mammal migrations in the Tarangire‐Manyara ecosystem and is often considered a key stepping stone and dispersal area for migratory herbivore species (Bond, Bradley, Kiffner, Morrison, & Lee, 2017; Kiffner, Nagar, Kollmar, & Kioko, 2016; Morrison & Bolger, 2014). At the onset of the wet season, wildebeest (*Connochaetes taurinus*), zebra (*Equus quagga*), and other large herbivores leave Tarangire National Park (TNP) and either move to the Simanjiro plains to the east of TNP or toward the northern plains near Lake Natron via MR (Lamprey, 1964; Morrison & Bolger, 2012). At the beginning of the dry season, migratory herbivores return from the nutrient‐rich wet season ranges in the north through MR to the Tarangire River in the core of TNP. In addition to this seasonal migration of grazing wildlife, some wildlife species (e.g., African elephants *Loxodonta africana* and Maasai giraffe *Giraffa camelopardalis*) occasionally move between Tarangire and Lake Manyara National Parks via MR (Lee & Bolger, 2017; Pittiglio, Skidmore, van Gils, & Prins, 2012).

### 2.2 Animal counts

We used line transect distance sampling (Thomas et al., 2010) to estimate densities of the most common wildlife and livestock species in MR (see Appendix S2 for scientific names of the considered species). From 2003 to 2008, MR management occasionally collected data on wildlife and livestock populations along road transects. Livestock was counted only intermittently in some of these surveys. Animals were counted along four driven transects that traversed the ranch and the perpendicular distance between the animal group and transect was assessed. Within a season, transects were occasionally repeated; repeated transects were considered as one transect, and sampling effort (line length) and sightings were combined accordingly (Buckland et al., 2001). Total line length ranged from 102 to 367 km in each seasonal survey.

**FIGURE 2:**
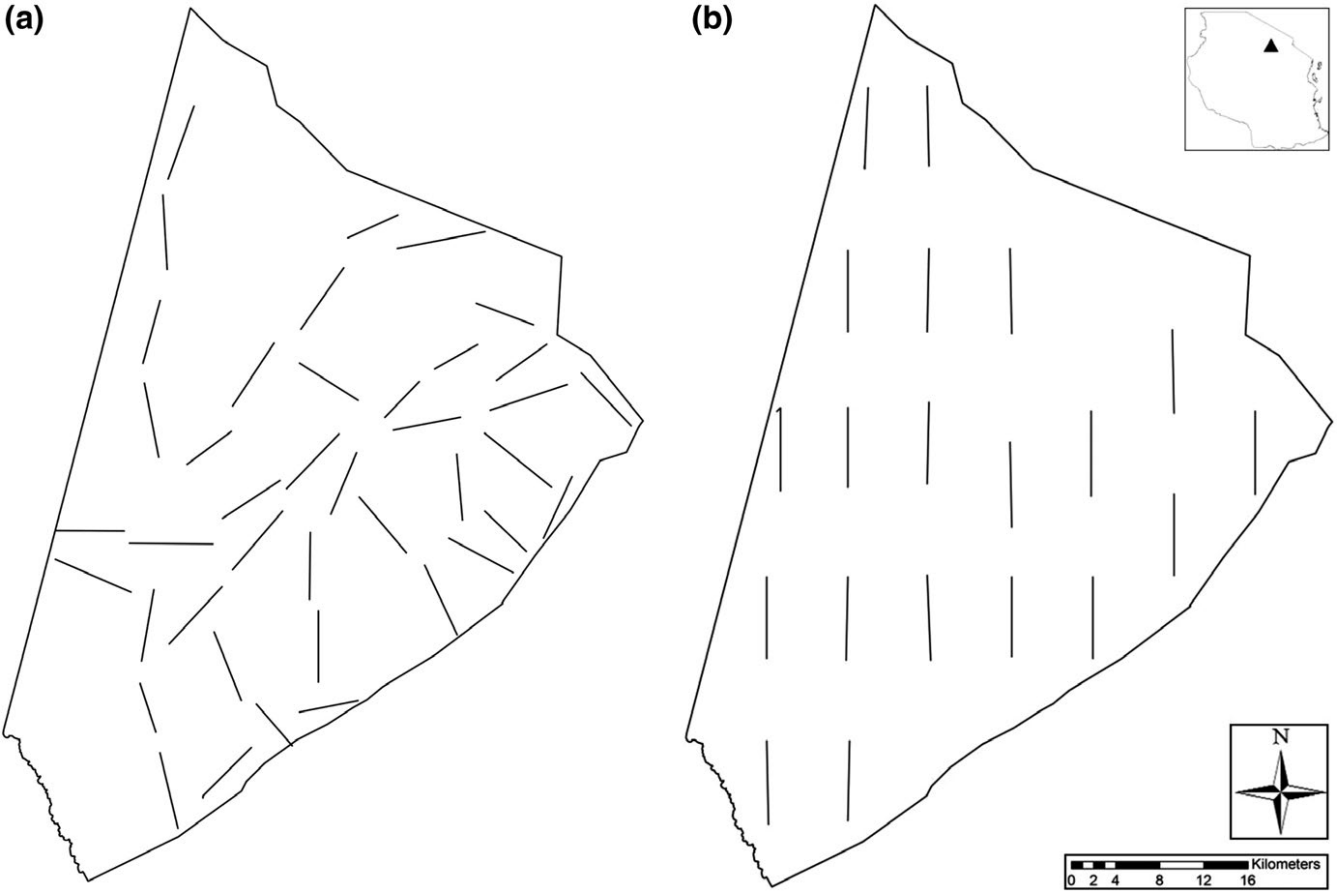
Map of Manyara Ranch showing (a) road transects and (b) systematic transects. The inset in the top right indicates the location of the study area within Tanzania

From late 2011 to late 2019, we carried out animal counts in each of the main seasons [long rains (LR): March–May; dry season (Dry): June–September; short rains (SR): October–December]. In 2019, we only conducted counts during LR and SR. We drove transects along minor roads and tracks that were distributed to cover the major habitats and regions of the ranch (Figure 2). Transects were usually 2 km in length and separated by 500m to ensure independence between transects and to prevent double counting of animals. If transects were repeated during one season, we combined effort and sightings accordingly. With some variation due to accessibility of the tracks and roads, we repeated the same transects (*n* = 32–47) in each of the seasons. Total line length per survey ranged from 62.9 to 198.53 km (the substantial variation in effort arises from repetitions of transects during some survey seasons). In total (2003–2019), we counted animals along *n* = 984 transects (2003–2008 *n* = 32; 2011–2019 *n* = 952), covering a total line length of 4,318 km (2003–2008:2,185 km; 2011–2019:2,133 km).

We drove transects at relatively slow speed (<20 km/h) and multiple observers (*n* = 3–7), including one MR ranger, spotted animals on both sides of the transect. Upon detection, we stopped the car and identified and counted animals. We measured the perpendicular distance between the animal (in case of animal groups: center of groups) directly in the field using a laser rangefinder (Bushnell Elite 1500). If animals moved away in response to the vehicle, we measured the perpendicular distance between their initial position and the transect line. Individuals of the same species within 50 m of each other were considered as one group.

### 2.3 Density estimation

We focused on the most frequently encountered species in MR. Because species‐specific detections were occasionally too few to model the detection function for each survey, we pooled all observations for one species across all surveys. We fitted two different detection functions in Distance 6.0, and for each model, we truncated 10% of the farthest sightings (Thomas et al., 2010). We fitted only half‐normal detection functions because this main function has been shown to produce unbiased estimates under most conditions (Prieto Gonzalez, Thomas, & Marques, 2017). Because we combined sightings from different surveys but detectability possibly varies by season (e.g., may be lower during the rainy season due to lusher vegetation compared to the dry season), we fitted one detection function in the conventional distance sampling framework (i.e., one detection function for the entire data per species) and one function in the multiple covariate distance sampling engine [defining season as a three‐level factor (long rains, dry season, and short rains) that could influence detectability of a species]. For each species, we selected the best fitting model among the two competing detection functions (CDS vs. MCDS with half‐normal detection function) using the AICc score (Buckland et al., 2001). Based on the selected detection function and the estimated herd size (setting the distance engine to estimate the mean of the observed cluster size of each season), we estimated season‐specific (using the poststratification option) density estimates including associated 95% confidence limits for each species. To describe overall patterns of population densities, we averaged the species‐specific point density estimates over the entire study period (2003–2019). To provide context to the derived density estimates, we compared species‐specific density estimates in MR with published density estimates from adjacent Lake Manyara and Tarangire National Parks and Mto wa Mbu Game Controlled Area (Kiffner et al., 2020; Kiffner, Hopper, & Kioko, 2016).

### 2.4 Validating density estimates

The roads that we used for our animal counts are not strictly systematically or randomly distributed across the ranch, and we thus implemented two independent line distance sampling surveys with 20 systematically distributed 2 km transects. Using two vehicles (each driving 10 transects), we conducted these counts one day prior to the road transect surveys of the 2018 and 2019 short rains. Density estimates from road and systematic survey designs are thus almost perfectly time‐matched. We placed start and end points of systematic transects on intersections of a 2 × 2 km grid that was overlaid the ranch. We shifted start (and end) points of a few transects because their initial trajectories ran through inaccessible terrain (Figure 2). We uploaded the GPS coordinates of start and end points, used the GPS to navigate, and drove in a straight line from start to end points with only slight deviations to avoid inaccessible terrain.

To test for differences between road and systematic transect designs, we estimated population densities of species with high encounter rates to allow modeling separate detection functions for each sampling regime (Buckland et al., 2001). These species included zebra (road: *n* = 58 observations; systematic: *n* = 53), wildebeest (road: *n* = 63; systematic: *n* = 42), Thomson's gazelle (road: *n* = 46; systematic: *n* = 59), and cattle (road: *n* = 57; systematic: *n* = 65). For each survey design, we combined sightings of 2018 and 2019 and estimated species‐ and year‐specific densities using the same specifications as during the main distance sampling analysis (half‐normal detection function, truncation of 10% of farthest distances; mean herd size option to estimate herd size, poststratification to produce year‐specific estimates). We used a *z*‐test to test for differences between time‐matched density estimates of the two sampling regimes (Buckland et al., 2001).

### 2.5 Trend assessments and correlational analysis

Our temporal trend analysis followed a stepwise analytical approach that has previously been used by other scholars (e.g., Barnes et al., 2016). To assess broad temporal trends in population densities, we fitted species‐specific generalized linear mixed models to the mean density estimates which were derived in the distance sampling framework. In line with common methods for trend analysis of wildlife population densities, these models were defined with a *log*‐link (Barnes et al., 2016; Thomas, 1996). To allow for *log*‐transformation, we replaced density estimates of “0” with “0.01.” To account for seasonal differences of animal densities and the repeated measures design, we defined the three‐level variable “season” (long rains, dry season, and short rains) as random effect in our models. As fixed effect, we considered the variable “year” and its regression coefficient indicates the relative annual change in density of target species. All models were fitted in the statistical software *R* (R Core Team, 2016) with the “*glmer*” function of the *lme4* package (Bates, Mächler, Bolker, & Walker, 2015). We are aware that this approach does not account for uncertainty associated with the density estimates (as it only uses the point density estimates), but this framework allowed us to assess whether animal populations were overall decreasing, increasing, or remaining constant over the study period after accounting for potential seasonal variation (Kiffner et al., 2020).

To test the hypothesis that competition between livestock and wildlife species results in observable and negative population‐level effects on wildlife species of similar feeding niches, we tested for nonparametric correlations (Kendall's correlation test) between time‐matched population densities of wildlife and livestock species. We restricted these analyses to the following wildlife–livestock pairs: densities of cattle versus densities of large, obligate grazing wildlife species (zebra, wildebeest, and waterbuck); densities of donkeys versus densities of large, obligate grazing wildlife species (zebra, wildebeest, and waterbuck); and densities of sheep and goats (combined) versus densities of medium‐sized mixed‐feeding wildlife (Grant's gazelle, impala, and Thomson's gazelle). To test the hypothesis that domestic dog population densities have negative and observable population‐level consequences for wildlife species with a similar ecological niche and for potential prey species, we tested for correlations between the density of domestic dogs and densities of black‐backed jackals, warthogs, and dik‐diks.

## 3 RESULTS

### 3.1 Validating density estimates

Density estimates of the four tested species (zebra, wildebeest, Thomson's gazelle, and cattle) from road transect surveys were lower than those derived from time‐matched systematically distributed transects. However, density estimates did not differ significantly (all *p*‐values > .09) across survey design in any of the surveys (Appendix S1). Thus, density estimates derived from road transects probably yield a conservative measure of animal densities (at least for the considered species). Because of the substantial overlap in density estimates (and thus uncertainty associated whether road transects cause actual undercounting bias of target species), we did not employ correction factors for the time series of density estimates.

### 3.2 Population density estimates

**FIGURE 3:**
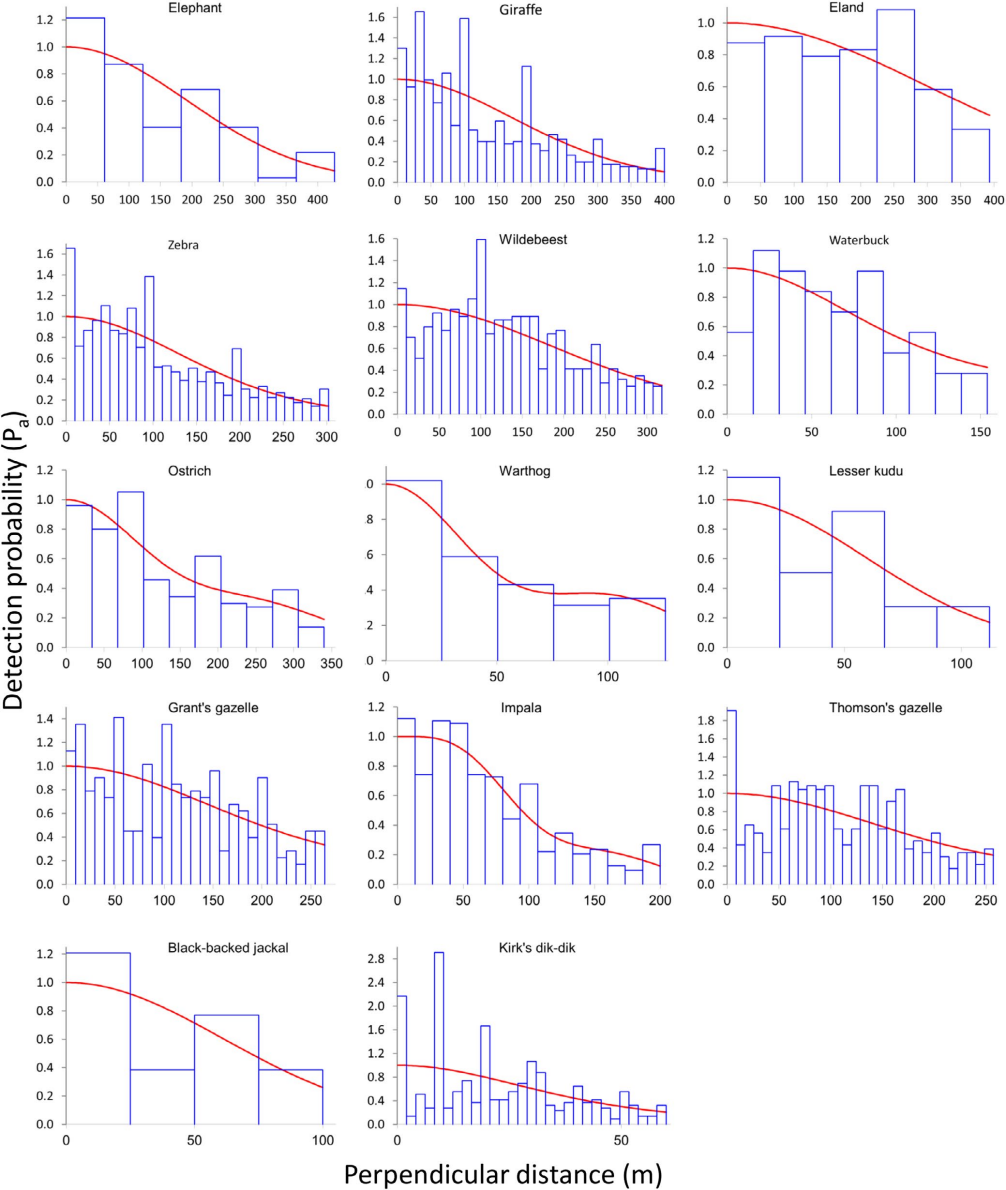
Detection functions of wildlife species based on data collected along road transects in Manyara Ranch (northern Tanzania) from 2003 to 2019. Histograms (blue bars) show the observed sighting frequencies, and the red lines describe the fitted detection functions

**FIGURE 4:**
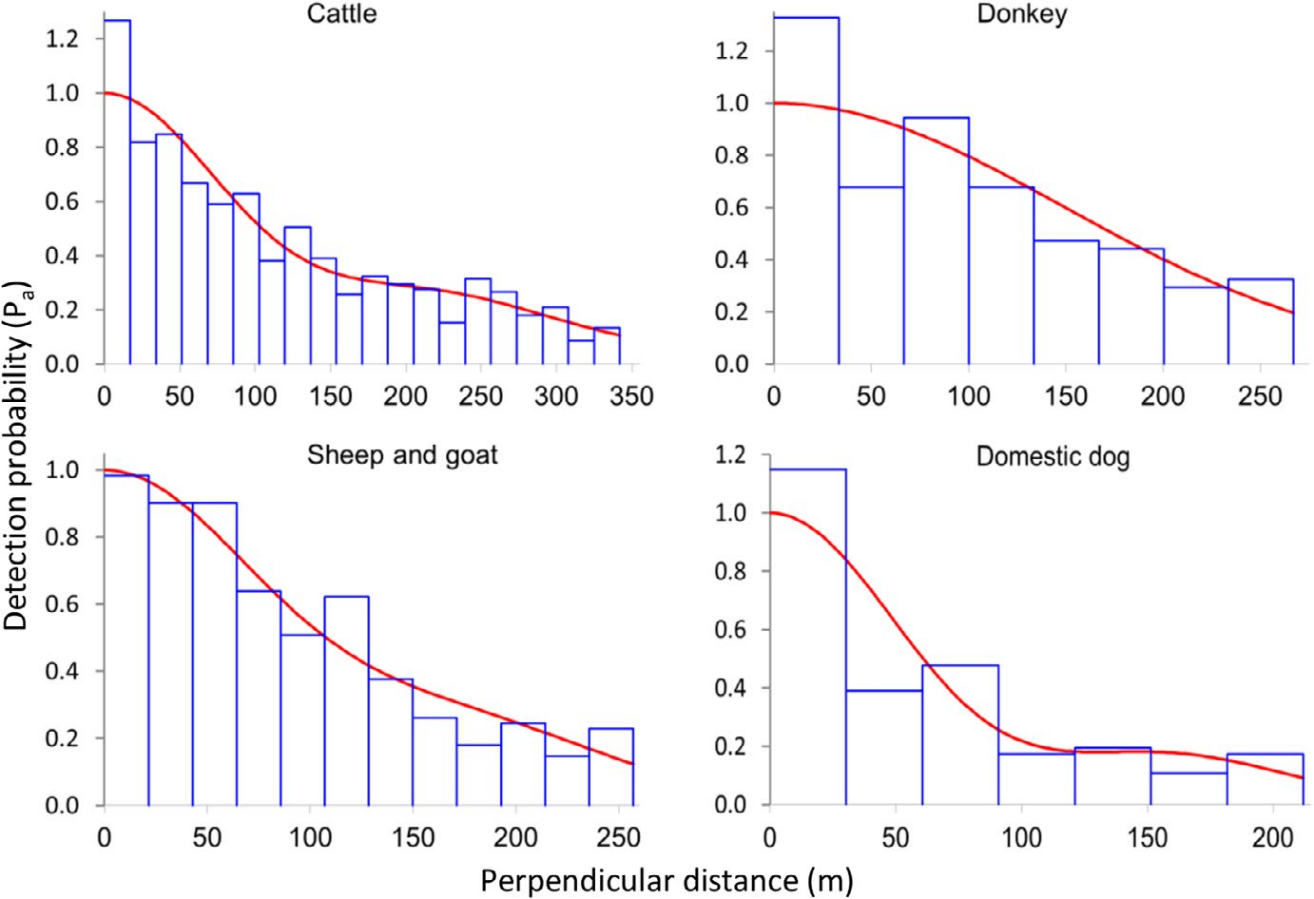
Detection functions of livestock species based on data collected along road transects in Manyara Ranch (northern Tanzania) from 2003 to 2019. Histograms (blue bars) show the observed sighting frequencies, and the red lines describe the fitted detection functions

We estimated seasonal population sizes of fourteen wildlife species and four livestock species (groups) (Appendix S2). In all but two species (waterbuck and black‐backed jackal), encounters exceeded the recommended minimum of 60 sightings to model detection functions (Buckland et al., 2001). For seven species (giraffe, zebra, wildebeest, waterbuck, Grant's and Thomson's gazelle, and dik‐dik), model selection suggested that seasonality affected detection probabilities, whereas conventional distance sampling models were selected for all other species (Appendix S2). As indicated by nonsignificant *p*‐values of *Χ* ^2^‐goodness‐of‐fit tests and visual assessments of fitted detection functions (Figure 3), detection functions for most (9 out of 14) wildlife species fit the data well. Detection functions of livestock mostly (3 out of 4) did not fit as well (Appendix S2), mostly due to a heaping of sightings near the transect line (Figure 4).

**FIGURE 5:**
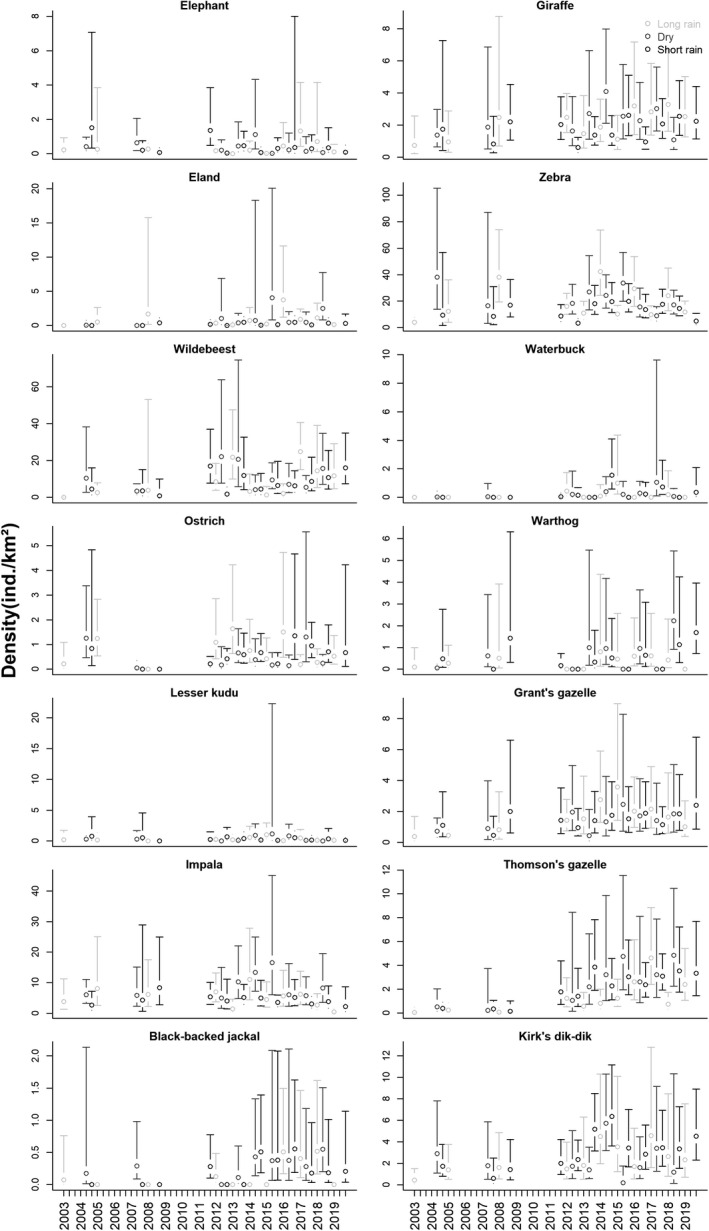
Estimated seasonal density estimates (open circles) and associated 95% confidence intervals (error bars) of 14 wildlife species in Manyara Ranch (northern Tanzania) from 2003 to 2019

Overall, the wildlife species assemblage was numerically dominated by two large and migrating grazers (Figure 5): zebra (mean density = 17.7 ind./km^2^; range of point estimates = 3.3–42.4) and wildebeest (8.9 ind./km^2^; range = 0–24.8). Average densities of other wildlife species were in descending order: impala (5.8 ind./km^2^; range = 0.4–16.5), Kirk's dik‐dik (2.6 ind./km^2^; range = 0.2–6.4), Maasai giraffe (2.0 ind./km^2^; range = 0.6–4.1), Thomson's gazelle (2.0 ind./km^2^; range = 0–4.8), Grant's gazelle (1.5 ind./km^2^; range = 0.4–3.6), eland (0.7 ind./km^2^; range = 0–4), ostrich (0.6 ind./km^2^; range = 0–1.6), warthog (0.5 ind./km^2^; range = 0–2.2), elephant (0.4 ind. km^2^; range = 0–1.5), lesser kudu (0.3 ind./km^2^; range = 0–1.2), waterbuck (0.2 ind./km^2^; range = 0–1.6), and black‐backed jackal (0.2 ind./km^2^; range = 0–0.6).

**FIGURE 6:**
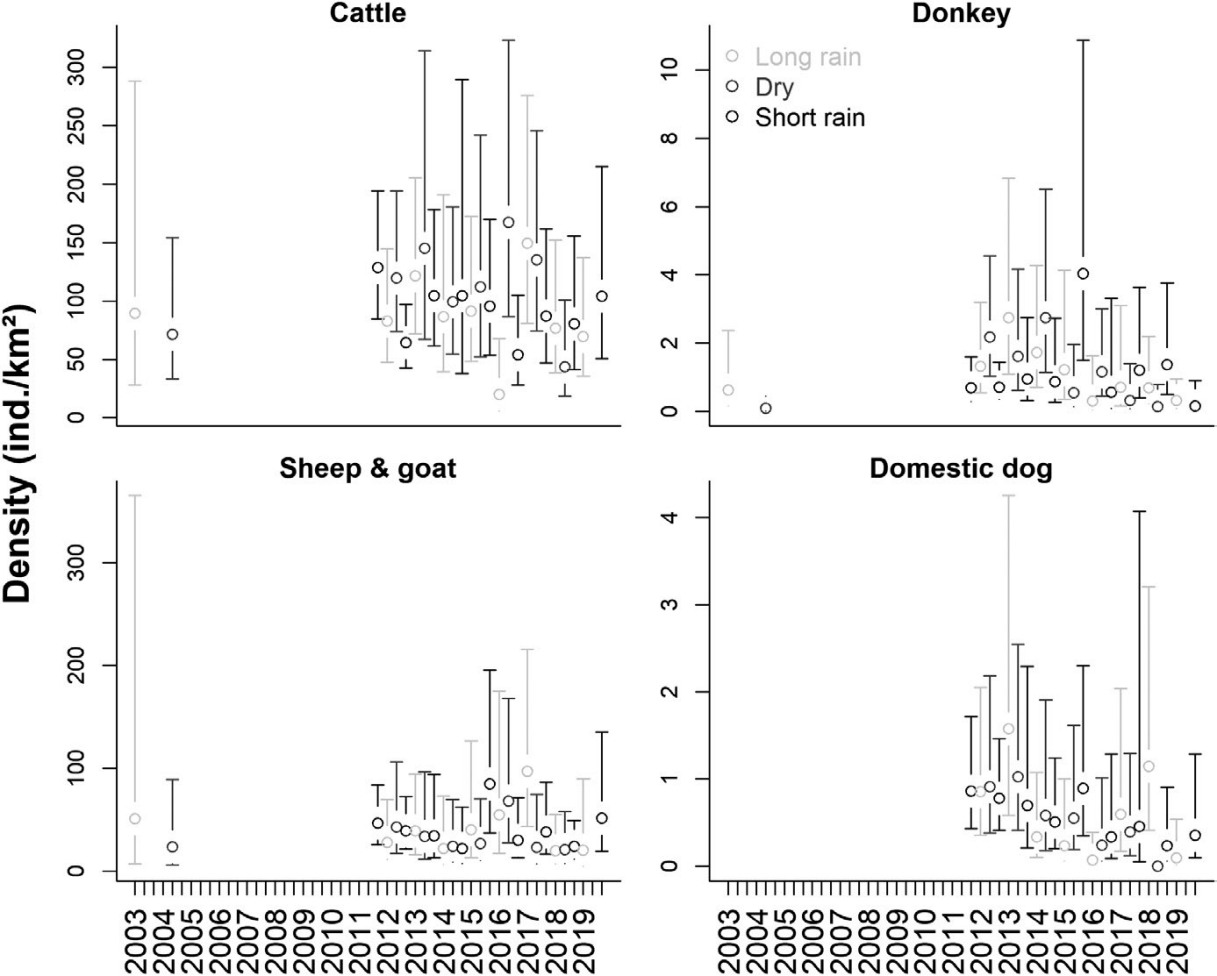
Estimated seasonal density estimates (open circles) and associated 95% confidence intervals (error bars) of livestock species (groups) in Manyara Ranch (northern Tanzania) from 2003 to 2019

Cattle (96.4 ind./km^2^; range = 19.9–167.3) and goat and sheep (38.8 ind./km^2^; range = 20.1–97.0) were the most abundant of all surveyed mammal species (Figure 6). The two other domestic species, donkey (1.1 ind./km^2^; range = 0.1–4.0) and domestic dog (0.6 ind./km^2^; range = 0–1.6), occurred at comparatively low densities.

Point estimates of both wildlife and livestock species were associated with relatively large margins of error, particularly during the 2003–2008 surveys (Figure 5 and Figure 6, and data uploaded to Dryad.org). In all species, the variation in encounter rates between transects contributed the most (mean: 68.8%; range: 50.9%–85.2%) to the width of the 95% confidence intervals (Appendix S3). Uncertainty associated with detection probabilities (mean: 8.6%; range: 1.1%–21.9%) and group sizes (mean: 22.6%; range: 8.8–35.1) contributed relatively little to overall precision of density estimates.

### 3.3 Population trends over time

Densities of five wildlife species (elephant, ostrich, warthog, lesser kudu, and Grant's gazelle) did not differ across the three main seasons (Appendix S4). Among the wildlife species that showed seasonal variation in densities, most species (8 out of 9) had greatest densities during the dry season, and intermediate densities during either the short rains (wildebeest, waterbuck, impala, Thomson's gazelle, black‐backed jackal), or the long rains (giraffe, eland, zebra). Kirk's dik‐dik was the only species that reached greatest densities during the short rains. None of the livestock species (groups) showed signs of seasonal variation in density estimates (Appendix S4).

Indicated by positive regression coefficients (and confidence intervals not overlapping with zero) of the variable “year,” the majority (8 out of 14) of wildlife species (giraffe, eland, wildebeest, waterbuck, Grant's and Thomson's gazelle, black‐backed jackal, and dik‐dik) showed positive annual density changes from 2003 to 2019 (Appendix S4). Confidence intervals of regression coefficients for the yearly change in density estimates in models for elephant, zebra, ostrich, warthog, lesser kudu, and impala overlapped with zero, indicating that populations of these species neither declined nor increased substantially over the study period.

Similarly, the models suggest that cattle, donkey and sheep and goat populations remained relatively constant over the study period (Appendix S4); please note that cattle, donkey, and goat and sheep were only counted during two surveys of the 2003–2008 surveys. The model for domestic dogs, however, suggests that their population densities declined substantially from 2011 to 2019 (domestic dogs were not counted during the 2003–2008 surveys).

### 3.4 Population‐level correlations between wildlife and livestock species

Population densities of obligate grazing wildlife species (zebra, wildebeest, and waterbuck) were not significantly correlated with densities of cattle (*p* ≥ .07) or donkeys (*p* ≥ .38) (Appendix S5). Similarly, population densities of mixed‐feeding wildlife species (Grant's gazelle, impala, Thomson's gazelle) were not significantly (*p* ≥ .54) associated with sheep and goat densities (Appendix S5). Domestic dog densities were not significantly correlated with dik‐dik and black‐backed jackal densities. However, domestic dog densities were significantly and negatively correlated with warthog densities (Appendix S5).

## 4 DISCUSSION

Results of our 17‐year wildlife monitoring program suggest that (a) MR supports relatively high densities of multiple herbivore species throughout the year, (b) wildlife densities were typically greatest during the dry season, (c) the surveyed wildlife populations remained constant or increased over time, (d) and there is little evidence for negative population‐level correlations between livestock and wildlife populations. In light of pervasive long‐term wildlife declines in East Africa (Craigie et al., 2010) and in fully protected areas of the Tarangire‐Manyara ecosystem (Kiffner et al., 2017; Morrison, Link, Newmark, Foley, & Bolger, 2016), this conservation model can possibly serve as a working example to conserve biodiversity, traditional livelihoods, and spatial connectivity in working landscapes (Western et al., 2020).

### 4.1 Conservation value of pastoral areas

**FIGURE 7:**
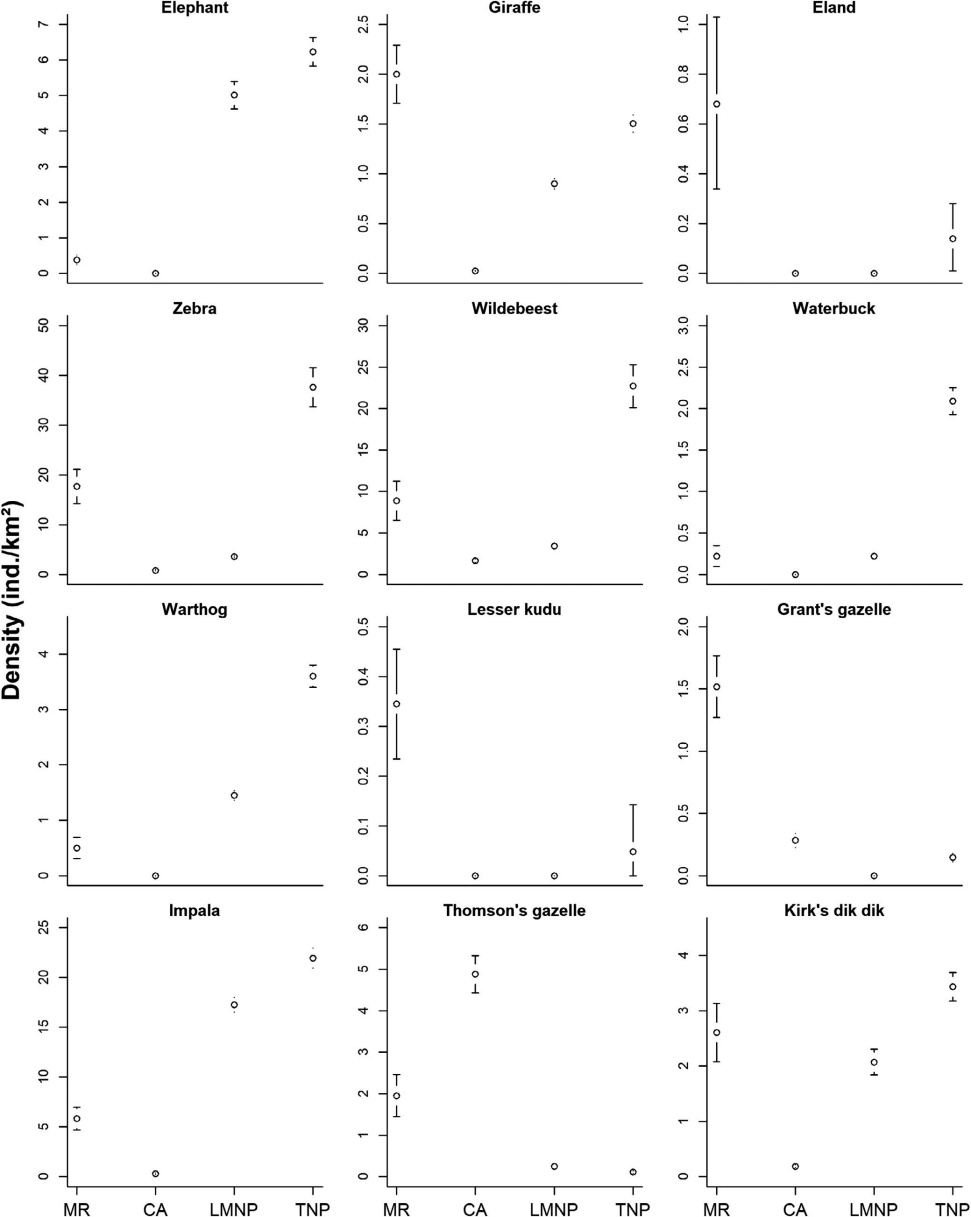
Estimated average densities (error bars denote 95% confidence intervals) of twelve (comparative data for ostrich and black‐backed jackal were not available for the other areas) wildlife species in Manyara Ranch (MR), adjacent Mto wa Mbu game controlled area (CA), Lake Manyara (LMNP) and Tarangire (TNP) National Parks. MR estimated are averages of the point estimates from 2003 to 2019 (this study), whereas estimates from CA, LMNP, and TNP are averages of point estimates from 2011 to 2018 (Kiffner et al., 2020). Eland and lesser kudu estimates from TNP were derived from a 2011–2014 dataset (Kiffner, Hopper, et al., 2016)

Beyond sustaining high large mammal species richness (Kiffner, Wenner, LaViolet, Yeh, & Kioko, 2015), MR supports densities of wildlife species that are broadly similar (zebra, wildebeest, waterbuck, and dik‐dik) or possibly even greater (giraffe, eland, lesser kudu, Grant's gazelle, and Thomson's gazelle) than densities in national parks (which do not permit livestock keeping) in the Tarangire‐Manyara ecosystem (Figure 7). However, densities of elephant, warthog, and impala were markedly lower in MR compared to nearby Lake Manyara and Tarangire National Parks, which may indicate that these species are potentially (directly or indirectly) negatively affected by livestock grazing and associated presence of herders and domestic dogs (Figure 7). Except for the Thomson's gazelle, wildlife densities in MR were substantially greater than those in the adjacent human‐dominated game controlled area (Figure 7 and see Kiffner, Nagar, et al., 2016). Overall, these comparisons of wildlife densities across protected areas highlight the complementary conservation value of different conservation approaches in this and other savannah ecosystems (Caro, Gardner, Stoner, Fitzherbert, & Davenport, 2009). Specifically, these comparisons suggest that some wildlife species (e.g., both gazelle species) can persist relatively well in livestock‐dominated areas (see also Bhola, Ogutu, Said, Piepho, & Olff, 2012; Georgiadis et al., 2007; Rannestad et al., 2006). However, densities of Thomson's gazelles in the Serengeti ecosystem are approximately 10× greater compared to MR (Dublin et al., 1990). In contrast, other wildlife species (e.g., elephants, warthogs) may only occur at relatively low densities in livestock‐dominated areas (Bhola et al., 2012; Ogutu et al., 2016; Treydte, Edwards, & Suter, 2005).

Although these spatial wildlife density comparisons provide some insights in the overall conservation value of MR, they do not provide causal inference for assessing the impact of livestock grazing on wildlife population densities. Most notably, the baseline of our monitoring was taken well after the commencement of livestock keeping in the area; clearly, this aspect may substantially underestimate possible impacts of livestock grazing on wildlife populations (Mahoub et al., 2017). Moreover, for some species the habitat quality in MR may be even more favorable than in adjacent protected areas: soil nutrient concentrations (Bond, Strauss, & Lee, 2016) and precipitation (due to its proximity to a Losimingori mountain and associated orographic rainfall) in MR are probably greater than in TNP, and, due to several dams, surface water is readily available during the dry season (Beattie, Olson, Kissui, Kirschbaum, & Kiffner, 2020). Therefore it may well be that MR provides greater forage quantity and quality as well as surface water availability to herbivores than adjacent protected areas.

Indeed, the persistence and comparable high densities of relatively rare antelope species in the ecosystem (e.g., eland, lesser kudu) suggests that MR provides suitable habitats for these species in the greater Tarangire‐Manyara ecosystem. Yet, their population sizes in MR are probably small [c. 125 (95% CI: 62–188) eland; c. 63 (43–83) lesser kudu] even when taking some possible (but unquantified) undercounting bias into account that may have been caused by the elusive behavior of these species (Kingdon, 1997). The trajectories of such small populations in MR are likely subject to stochastic events (Caughley, 1994), and their long‐term population viability in the ecosystem is likely dependent on dispersal from and to different protected areas.

Our multi‐species and long‐term monitoring data suggest that most species aggregate in MR during the dry season (Appendix S4). Especially the few man‐made dams probably enable the dry season usage of MR by water‐dependent wildlife species such as zebra, wildebeest, and waterbuck (Veldhuis, Kihwele, et al., 2019). However, during the dry season, surface water is heterogeneously distributed in the ranch (Beattie et al., 2020), which may facilitate the coexistence of both water‐dependent and less‐water‐dependent wildlife as well as livestock species in MR (Veldhuis, Kihwele, et al., 2019).

MR provides not only key habitats for resident herbivores year‐round but is also a vital stepping stone for the ungulate migration in the ecosystem (Bolger, Newmark, Morrison, & Doak, 2008; Bond et al., 2017; Lamprey, 1964). Interestingly, the migratory wildebeest and zebra populations in MR reach greater densities than the (mostly) nonmigratory populations in LMNP (Figure 7), probably because they are able to better track the seasonal variation in food resources (Morrison et al., 2016; Voeten et al., 2010). Evidence from Botswana suggests that blocking migration corridors can cause sharp population declines of grazing ungulates in ecosystems (Mbaiwa & Mbaiwa, 2006; Spinage, 1992). Relatively large, unfenced rangelands such as MR can maintain habitat for herbivores (and thus effectively increase range sizes of these species) and ensure animal movement across the wider landscape. Maintaining this landscape connectivity for migratory wildlife species, for dispersal of nonmigratory species, and for pastoralists and their livestock should thus be a high priority in this and other savannah ecosystems so that wildlife and livestock species can track the dynamic distribution of key resources (mainly forage and surface water) in the landscape (Bond et al., 2017; Durant et al., 2015). Conserving this connectivity for wide‐ranging wildlife depends on multiple stakeholders (Bolger et al., 2008; Harris, Thirgood, Hopcraft, Cromsight, & Berger, 2009) and ideally employs integrated landscape planning approaches. Tanzania is currently aiming to formally protect its remaining wildlife corridors (Caro, Jones, & Davenport, 2009; Riggio & Caro, 2017); strategically placed, unfenced pastoral areas with effective anti‐poaching enforcement can possibly be a practical option to enable wildlife movement between key protected areas while simultaneously supporting the traditional lifestyle of pastoralist ethnicities (Durant et al., 2015; Western et al., 2020).

### 4.2 Managing livestock–wildlife coexistence

Clearly, direct and indirect interactions with livestock can be detrimental for wildlife populations even though our cross‐species correlation analyses did not provide strong evidence for population‐level consequences of interference competition between cattle, donkeys, sheep, goats, and wildlife species (Appendix S5). For example, overgrazing by livestock is locally apparent, particularly in the edge areas of MR (Kissui & Kioko, 2013), and may lower the primary productivity in these locations. On the other hand, cattle may be at risk of acquiring the virus that causes malignant catarrhal fever during the wildebeest calving season in the wet season (Lankester et al., 2015). Furthermore, harassment of wildlife by people (Kiffner et al., 2014) and their livestock‐guarding dogs (Appendix S5) may have negative consequences for certain wildlife species (Doherty et al., 2017). In particular, investigating and quantifying the direct (predation) and indirect (risk) effects of domestic dogs on warthog populations may be worthwhile.

However, evidenced by this long‐term monitoring, the livestock–wildlife coexistence model in MR appears to be effective in supporting a species‐rich and relatively high‐density wildlife assemblage (Figure 5; Figure 6; Appendix S3). Although difficult to test quantitatively, the main contributing factors toward constant and increasing wildlife population densities are likely (a) effective anti‐poaching efforts and (b) spatio‐temporal grazing restrictions. While the grazing restrictions are not fully effective (as evidenced by no substantial seasonal differences in livestock densities although livestock are not supposed to use MR during the rains; Appendix S3), livestock are mostly kept out of the core area of the ranch. Likely, this grazing regime provides a spatial refuge for wildlife species and a reserve of forage during the dry season in the core area (Durant et al., 2015; Vanak et al., 2013) and helps maintaining short grass areas (which may provide suitable habitat for species such as wildebeest and Thomson's and Grant's gazelles) in some of the peripheral areas (Bhola et al., 2012). Theoretically, strategically planning livestock grazing can increase biodiversity outcomes if livestock grazing is managed to minimize competition with wildlife, and to maximize heterogeneity of grasslands, creation of nutrient hotspots and facilitation of high‐quality grazing (Fynn et al., 2016). However, enforcing such grazing rules and gaining community support for the adherence to these rules requires substantial and constant efforts.

Further testing of the various impacts of livestock grazing, for example by using spatially explicit, wildlife and livestock distribution models (Schuette et al., 2016) or experimental studies (Goheen et al., 2018), may provide additional insights into mechanisms governing livestock–wildlife coexistence in rangelands. While the temporal and spatial extent of livestock grazing can clearly affect the impact of livestock grazing on rangelands and wildlife populations (Fynn et al., 2016), the ability of rangelands to maintain habitat heterogeneity and to provide sufficient refuge areas for wildlife is also a function of livestock densities in relation to primary productivity (Kowal et al., 2019). Additional research aimed at finding tipping points between livestock densities and wildlife persistence may provide further guidelines for rangeland management that supports goals of livestock production and wildlife conservation.

In addition to understanding and managing the indirect and direct impacts of livestock grazing, good relationships between MR management and adjacent communities are key to ensuring effective wildlife conservation in multiple‐use areas. Contradictory messages and unclear communication in the process of the Manyara Ranch land trust establishment in the early 2000s created negative attitudes toward MR among adjacent communities in the past (Goldman, 2011). The ranch currently sustains substantial efforts in improving community relationships through stakeholder meetings, employment of staff (herders and game scouts) from adjacent communities, revenue sharing programs, and livestock breeding programs, and consequentially, community attitudes appear to have improved. One persisting conflict is livestock depredation by lions (*Panthera leo*) near water points during the dry season and the subsequent retaliatory killing of the lions by *Maasai* herders. Although it may not always be possible to find win‐win solutions in such high tension situations, areas of high livestock depredation risk by lions can be predicted relatively reliably (Beattie et al., 2020). In turn, this information can be used to guide livestock grazing away from high‐risk areas to reduce livestock depredation events.

### 4.3 Wildlife monitoring in pastoral areas

To assess the long‐term dynamics of rangelands, wildlife monitoring is an essential but rarely implemented tool in coupled livestock–wildlife systems (but see Georgiadis et al., 2007; Ogutu et al., 2016). Often, wildlife densities in East Africa are assessed using sample‐based aerial surveys (Ogutu et al., 2016; Stoner et al., 2007) but these surveys often fail to detect smaller‐bodied species (Greene et al., 2017; Jachmann, 2002) – species that often cope relatively well in human‐ and livestock‐dominated areas (Crees, Turvey, Freeman, & Carbone, 2019; Riggio et al., 2018). Ground‐based surveys may therefore provide a more suitable option to assess wildlife and livestock densities over time. In this specific case, the use of road transects resulted in undercounting bias (average: 60%; range: 36%–86%) compared to systematically distributed transects, yet the design‐based differences in population density estimates were not significantly different (Appendix S1). Thus, the use of road transects may be a cost‐effective method to monitor wildlife and livestock species (Caro, 2016). In other cases, however, road transects may yield substantially greater bias of density estimates compared to other, possibly more adequate methods (e.g., see elephant density comparison in Kiffner et al., 2017). Therefore, if road transects are utilized for regular wildlife population monitoring (e.g., if walking transects are prohibitive in costs or off‐road driving is not permitted or not possible), associated bias is ideally quantified empirically.

Overall, density estimates of many species were highly variable over the monitoring period (Figure 5; Figure 6). To some extent, the migratory nature of many abundant species (e.g., zebra and wildebeest) may explain some of the observed variation in density estimates between surveys. Despite relatively high sampling effort, confidence intervals of density estimates were relatively wide, particularly during the early monitoring phase when few, but long transects were driven (Figure 5; Figure 6). Precision of the estimates can be improved by increasing the number of transects (Buckland et al., 2001), but the clumped distribution of many wildlife species creates large variation in encounter rates between transects (Appendix S3) which causes wide margins of errors (Fewster et al., 2009; Ogutu et al., 2006). To increase the precision of estimates (and thus to possibly increase the power to detect changes over time), stratified sampling or spatially explicit distance sampling models may be promising tools to describe and analyze temporal trends of the wildlife assemblage in greater detail (Barabesi & Fattorini, 2013; Miller, Burt, Rexstad, & Thomas, 2013; Oedekoven, Buckland, Mackenzie, Evans, & Burger, 2013).

### 4.4 Conclusion

The ongoing human population growth in East Africa limits options to expand the extent of fully protected areas (Caro & Davenport, 2016), which urges conservationists to develop, test, and implement human–wildlife coexistence models to counteract the biodiversity crisis (Crego et al., 2020; Tyrrell, du Toit, & Macdonald, 2020; Western et al., 2020). Our case study shows that wildlife populations in a managed pastoral area remained constant over a 17‐year span. This provides further evidence that wildlife–livestock coexistence is possible in East African rangelands (Georgiadis et al., 2007; Kinnaird & O’Brien, 2012; Schuette et al., 2016), provided that seasonal, spatial, and numerical restrictions on livestock use are implemented (Tyrrell, Russell, & Western, 2017) and poaching is effectively controlled.

## CONFLICT OF INTEREST

None declared.

## AUTHOR CONTRIBUTIONS

**Christian Kiffner:** Conceptualization (lead); data curation (lead); formal analysis (lead); funding acquisition (supporting); investigation (lead); methodology (lead); project administration (lead); supervision (lead); visualization (lead); writing – original draft (lead). **John Kioko:** Conceptualization (supporting); data curation (supporting); investigation (equal); supervision (supporting); visualization (supporting); writing – review and editing (supporting). **Jack Baylis:** Data curation (supporting); formal analysis (supporting); investigation (supporting); writing – review and editing (supporting). **Camille Beckwith:** Data curation (supporting); formal analysis (supporting); investigation (supporting); writing – review and editing (supporting). **Craig Brunner:** Data curation (supporting); formal analysis (supporting); investigation (supporting); writing – review and editing (supporting). **Christine Burns:** Data curation (supporting); formal analysis (supporting); investigation (supporting); writing – review and editing (supporting). **Vasco Chavez‐Molina:** Data curation (supporting); formal analysis (supporting); investigation (supporting); writing – review and editing (supporting). **Sara Cotton:** Data curation (supporting); formal analysis (supporting); investigation (supporting); writing – review and editing (supporting). **Laura Glazik:** Data curation (supporting); formal analysis (supporting); investigation (supporting); writing – review and editing (supporting). **Ellen Loftis:** Data curation (supporting); formal analysis (supporting); investigation (supporting); writing – review and editing (supporting). **Megan Moran:** Data curation (supporting); formal analysis (supporting); investigation (supporting); writing – review and editing (supporting). **Caitlin O'Neill:** Data curation (supporting); formal analysis (supporting); investigation (supporting); writing – review and editing (supporting). **Ole Theisinger:** Data curation (supporting); investigation (supporting); supervision (supporting); writing – review and editing (supporting). **Bernard Kissui:** Conceptualization (supporting); data curation (equal); funding acquisition (lead); investigation (equal); project administration (supporting); supervision (supporting); writing – review and editing (supporting).

## Supporting information

Appendix S1‐S5

## Data Availability

Seasonal density estimates and associated confidence intervals of MR and averaged density estimates (incl. 95% confidence intervals) of protected area in the Tarangire‐Manyara ecosystem (Figure 7) are accessible at https://doi.org/10.5061/dryad.zgmsbcc8c.
