# Supplementary material for: Long‐term persistence of wildlife populations in a pastoral area
Source: Ecol Evol. 2020 Aug 7;10(18):10000–16. doi: 10.1002/ece3.6658 (PMC7520174; doi:10.1002/ece3.6658)
Supplement: Supplementary file 1 — Appendix S1‐S5 [file ECE3-10-10000-s001.docx]

# Appendix

**S 1.** Density estimates and associated coefficients of variation (CV) of frequently encountered species (zebra, wildebeest, Thomson’s gazelle and cattle) derived from road surveys (‘Road’) and systematically distributed transects (‘Systematic’) in Manyara Ranch (northern Tanzania) conducted during the 2018 and 2019 short rain seasons. The associated *z*-test statistics (‘*z*’) and *p*-values indicate no significant differences between the two transect designs.

| **Species-year comparison** | **Road** | | **Systematic** | |  |  |
| --- | --- | --- | --- | --- | --- | --- |
|  | **D** | **CV** | **D** | **CV** | ***z*** | ***p*-value** |
| Zebra 2018 | 16.97 | 34.01 | 19.68 | 37.98 | -0.29 | 0.849 |
| Zebra 2019 | 4.15 | 38.45 | 8.47 | 35.29 | -1.27 | 0.204 |
| Wildebeest 2018 | 18.09 | 40.82 | 29.90 | 69.11 | -0.54 | 0.589 |
| Wildebeest 2019 | 12.89 | 41.20 | 18.43 | 45.12 | -0.56 | 0.575 |
| Thomson’s gazelle 2018 | 6.57 | 54.35 | 9.63 | 48.68 | -0.52 | 0.603 |
| Thomson’s gazelle 2019 | 2.98 | 40.54 | 8.13 | 34.39 | -1.69 | 0.091 |
| Cattle 2018 | 46.30 | 41.13 | 115.52 | 43.23 | -1.30 | 0.194 |
| Cattle 2019 | 67.22 | 37.45 | 92.88 | 40.92 | -0.56 | 0.575 |

**S 2.** Key parameters associated with selected half-normal detection functions used to estimate population densities of wildlife and livestock species in Manyara Ranch (northern Tanzania) based on road transect surveys conducted from 2003-2019. ‘n’ indicates the number of detections, ‘P_a_’ the estimated detection probability, ‘Lower and upper P_a_ CI’ the associated 95%-confidence intervals, ‘ESW’ is the estimated strip width in meters and its associated confidence intervals [‘Upper and lower ESW CI (m)’], and ‘*p*-value’ is the probability of the chi-squared goodness of fit-test. We selected between conventional (CDS) and multiple covariate distance sampling (MCDS) models with half-normal detection functions based on AICc scores.

| *Wildlife species* | **Scientific name** | **n** | **P_a_** | **Lower P_a_ CI** | **Upper P_a_ CI** | **ESW (m)** | **Lower ESW CI (m)** | **Upper ESW CI (m)** | ***p*-value** | **Model** |
| --- | --- | --- | --- | --- | --- | --- | --- | --- | --- | --- |
| Elephant | *Loxodonta africana* | 123 | 0.55 | 0.48 | 0.63 | 234 | 204 | 267 | 0.422 | CDS |
| Giraffe | *Giraffa tippelskirchi* | 750 | 0.55 | 0.52 | 0.58 | 221 | 210 | 232 | <0.001 | MCDS |
| Eland | *Taurotragus oryx* | 130 | 0.77 | 0.64 | 0.93 | 304 | 253 | 365 | 0.669 | CDS |
| Zebra | *Equus quagga* | 1460 | 0.57 | 0.55 | 0.59 | 173 | 167 | 179 | 0.001 | MCDS |
| Wildebeest | *Connochaetes taurinus* | 641 | 0.68 | 0.65 | 0.71 | 216 | 207 | 226 | 0.024 | MCDS |
| Waterbuck | *Kobus ellipsiprymnus* | 48 | 0.67 | 0.55 | 0.82 | 103 | 85 | 126 | 0.756 | MCDS |
| Ostrich | *Struthio camelus* | 233 | 0.53 | 0.45 | 0.64 | 181 | 152 | 217 | 0.463 | CDS |
| Warthog | *Phacochoerus africanus* | 69 | 0.54 | 0.39 | 0.75 | 68 | 49 | 94 | 0.846 | CDS |
| Lesser kudu | *Tragelaphus imberbis* | 68 | 0.63 | 0.51 | 0.77 | 70 | 57 | 86 | 0.390 | CDS |
| Grant's gazelle | *Nanger granti* | 337 | 0.70 | 0.66 | 0.75 | 186 | 175 | 198 | 0.356 | MCDS |
| Impala | *Aepyceros melampus* | 516 | 0.54 | 0.47 | 0.63 | 109 | 93 | 126 | 0.077 | CDS |
| Thomson's gazelle | *Eudorcas thomsonii* | 482 | 0.70 | 0.66 | 0.73 | 179 | 170 | 189 | 0.005 | MCDS |
| Black-backed jackal | *Canis mesomelas* | 50 | 0.69 | 0.54 | 0.87 | 69 | 54 | 87 | 0.078 | CDS |
| Dik-dik | *Madoqua kirkii* | 386 | 0.61 | 0.58 | 0.66 | 37 | 35 | 39 | 0.004 | MCDS |
| *Livestock species* |  |  |  |  |  |  |  |  |  |  |
| Cattle | *Bos* spp. | 902 | 0.43 | 0.39 | 0.47 | 147 | 134 | 161 | <0.001 | CDS |
| Donkey | *Equus africanus* | 175 | 0.65 | 0.57 | 0.73 | 172 | 152 | 195 | 0.032 | CDS |
| Sheep and goat | *Capra* spp. and *Ovis* spp. | 366 | 0.50 | 0.44 | 0.57 | 129 | 112 | 147 | 0.217 | CDS |
| Domestic dog | *Canis lupus familiaris* | 123 | 0.38 | 0.32 | 0.46 | 81 | 67 | 97 | 0.008 | CDS |

**S 3.** Component percentages of variation in density estimates of fourteen wildlife and four livestock species (group) in Manyara Ranch (northern Tanzania) associated with detection probability, encounter rates, and group sizes.

|  | **Detection probability** | **Encounter rate** | **Cluster size** |
| --- | --- | --- | --- |
| *Wildlife - average* | *8.6* | *68.8* | *22.6* |
| Elephant | 1.1 | 79.3 | 19.6 |
| Giraffe | 14.5 | 58.2 | 27.3 |
| Eland | 1.9 | 66.5 | 31.6 |
| Zebra | 10.7 | 65.4 | 23.9 |
| Wildebeest | 8.0 | 50.9 | 41.1 |
| Waterbuck | 21.9 | 67.6 | 10.5 |
| Ostrich | 3.0 | 71.3 | 25.6 |
| Warthog | 5.4 | 75.6 | 19.0 |
| Lesser kudu | 1.8 | 85.2 | 13.0 |
| Grant's gazelle | 13.0 | 59.9 | 27.1 |
| Impala | 3.6 | 61.3 | 35.1 |
| Thomson's gazelle | 10.8 | 67.1 | 22.1 |
| Black-backed jackal | 2.9 | 85.1 | 12.0 |
| Dik-dik | 21.3 | 69.9 | 8.8 |
|  |  |  |  |
| *Livestock - average* | *2.0* | *73.0* | *25.0* |
| Cattle | 2.1 | 63.3 | 34.7 |
| Donley | 1.3 | 76.5 | 22.1 |
| Sheep and goat | 2.0 | 70.1 | 27.9 |
| Domestic dog | 2.7 | 82.0 | 15.3 |

**S 4.** Regression coefficient estimates and associated 95%-confidence intervals (lower and upper CI) of general linear mixed models testing the effects of the linear predictor ‘year’ on estimated population densities of wildlife and livestock species in Manyara Ranch (northern Tanzania) from 2003-2019. Effects sizes that are non-overlapping with zero are highlighted in bold. The intercept of the mixed effect ‘season’ (long rains, dry season, and short rains) is presented in the three rightmost columns.

| *Wildlife* | Intercept | Lower CI | Upper CI |  | Year | Lower CI | Upper CI |  | Long rain | Dry | Short rain |
| --- | --- | --- | --- | --- | --- | --- | --- | --- | --- | --- | --- |
| Elephant | 90.800 | -89.553 | 271.152 |  | -0.046 | -0.135 | 0.044 |  | 0 | 0 | 0 |
| Giraffe | -89.430 | -154.566 | -24.333 |  | **0.045** | **0.012** | **0.077** |  | 0.008 | 0.035 | -0.043 |
| Eland | -397.222 | -609.169 | -185.032 |  | **0.197** | **0.091** | **0.302** |  | 0.340 | 0.364 | -0.704 |
| Zebra | -17.602 | -108.652 | 73.260 |  | 0.010 | -0.035 | 0.055 |  | 0.012 | 0.164 | -0.176 |
| Wildebeest | -300.202 | -484.932 | -116.750 |  | **0.150** | **0.059** | **0.242** |  | -0.137 | 0.125 | 0.011 |
| Waterbuck | -307.124 | -556.959 | -58.811 |  | **0.151** | **0.028** | **0.275** |  | -0.152 | 0.175 | -0.022 |
| Ostrich | -183.461 | -388.005 | 21.082 |  | 0.091 | -0.011 | 0.192 |  | 0 | 0 | 0 |
| Warthog | -50.898 | -356.766 | 254.970 |  | 0.024 | -0.128 | 0.176 |  | 0 | 0 | 0 |
| Lesser kudu | 3.516 | -204.978 | 212.010 |  | -0.003 | -0.106 | 0.101 |  | 0 | 0 | 0 |
| Grant's gazelle | -151.982 | -216.516 | -87.447 |  | **0.076** | **0.044** | **0.108** |  | 0 | 0 | 0 |
| Impala | 56.570 | -39.056 | 151.644 |  | -0.027 | -0.075 | 0.020 |  | -0.176 | 0.322 | -0.146 |
| Thomson's gazelle | -449.143 | -548.289 | -350.490 |  | **0.223** | **0.174** | **0.272** |  | -0.513 | 0.319 | 0.194 |
| Black-backed jackal | -310.944 | -540.478 | -83.357 |  | **0.153** | **0.040** | **0.267** |  | -0.408 | 0.502 | -0.095 |
| Dik-dik | -123.870 | -227.940 | -19.751 |  | **0.062** | **0.010** | **0.114** |  | -0.009 | -0.039 | 0.048 |
| *Livestock* |  |  |  |  |  |  |  |  |  |  |  |
| Cattle | 24.198 | -65.491 | 113.886 |  | -0.010 | -0.054 | 0.035 |  | 0 | 0 | 0 |
| Donkey | -1.423 | -192.426 | 189.580 |  | 0.001 | -0.094 | 0.095 |  | 0 | 0 | 0 |
| Sheep and goat | 19.122 | -72.142 | 110.386 |  | -0.008 | -0.053 | 0.038 |  | 0 | 0 | 0 |
| Domestic dog | 504.754 | 187.753 | 821.755 |  | **-0.251** | **-0.408** | **-0.094** |  | 0 | 0 | 0 |

**S 5.** Results of Kendalls correlation test (Τ=correlation coefficient) between the densities of selected livestock and wildlife species in Manyara Ranch (northern Tanzania) from 2003-2019.

|  | Cattle | | Donkey | | Sheep and goat | | Domestic dog | |
| --- | --- | --- | --- | --- | --- | --- | --- | --- |
|  | Τ | p-value | Τ | p-value | Τ | p-value | Τ | p-value |
| Zebra | -0.077 | 0.600 | 0.126 | 0.382 |  |  |  |  |
| Wildebeest | 0.255 | 0.070 | 0.015 | 0.931 |  |  |  |  |
| Waterbuck | 0.062 | 0.668 | -0.016 | 0.910 |  |  |  |  |
| Impala |  |  |  |  | -0.089 | 0.541 |  |  |
| Grant's gazelle |  |  |  |  | 0.089 | 0.541 |  |  |
| Thomson's gazelle |  |  |  |  | 0.028 | 0.862 |  |  |
| Warthog |  |  |  |  |  |  | **-0.323** | **0.034** |
| Dik-dik |  |  |  |  |  |  | -0.011 | 0.941 |
| Black-backed jackal |  |  |  |  |  |  | -0.121 | 0.421 |
